# Experimental Biology and Medicine: a global journal with rigorous publication standards

**DOI:** 10.3389/ebm.2024.10346

**Published:** 2024-09-10

**Authors:** Steven R. Goodman

**Affiliations:** Department of Pediatrics, University of Tennessee Health Science Center (UTHSC), Memphis, TN, United States

**Keywords:** biomedical, journal, global, review process, paper mills

Roughly 10 months ago the Society of Experimental Biology and Medicine (SEBM) Council approved its journal *Experimental Biology and Medicine* (EBM) opening an office on the continent of Australia/Oceania, with Sulev Koks, MD/PhD at Murdoch University becoming our new Global Editor from this continent. With the opening of the Australia/Oceania office, and the existing offices in the United States, Taiwan, China, the United Kingdom, Brazil, and Ghana, EBM now has offices and Global Editors on all six habitable continents. We also have twenty-two scientific categories covering the breadth of modern biomedical research and all Translational stages from T0 to T4 spanning basic, translational, clinical, clinical trial and population research.

Exactly 2 weeks after our addition of the Australia/Oceania office, we began accepting manuscripts though the Frontiers portal on 4th November 2023, with our first papers accepted for publication in January 2024. We have used the same rigorous single blind review process that EBM has been known for since our first published issue in 1904. Our standards remain the same and our acceptance rate since changing publishers to Frontiers in 2024 has been 18%, which is lower than the acceptance rate with our prior publisher which ranged from 20% to 30% annually. We have added to our workflow rigorous checks and standard operating procedures to screen out any manuscripts that have the possibility of being generated by a paper mill, which has become a common pervasive problem for all biomedical research journals. We have gone beyond typical procedures by having our new Deputy Editor Nicola Conran, PhD prescreening manuscripts with paper mill characteristics and checking raw data supplied by authors when deemed necessary. We go through this stringent process to assure our readers and ourselves that manuscripts accepted for publication in EBM can be trusted to be honest and important contributions to advancing biomedical research. If we find any of our published articles that do not meet these standards due to any form of research misconduct, they are swiftly retracted.

As Editor-in-Chief of EBM for over 18 years, you can be assured that bringing the research community important and honest contributions to the scientific record is always foremost on my mind. In my academic university career, I have served as a Research Integrity Officer, a Vice President for Research, and a Vice Chancellor for Research. In each of these roles, I was overseeing all research integrity for my Academic Health Center. I believe that this long overlapping experience in dealing with Research misconduct issues at Academic Institutions and journal publications, places EBM in a unique position to be worthy of your trust.

